# Supplementary material for: Combining Time-Restricted Wheel Running and Feeding During the Light Phase Increases Running Intensity Under High-Fat Diet Conditions Without Altering the Total Amount of Daily Running
Source: Int J Mol Sci. 2025 Aug 7;26(15):7658. doi: 10.3390/ijms26157658 (PMC12347854; doi:10.3390/ijms26157658)
Supplement: Supplementary file 1 [file ijms-26-07658-s001.zip › ijms-3715755-supplementary.pdf]

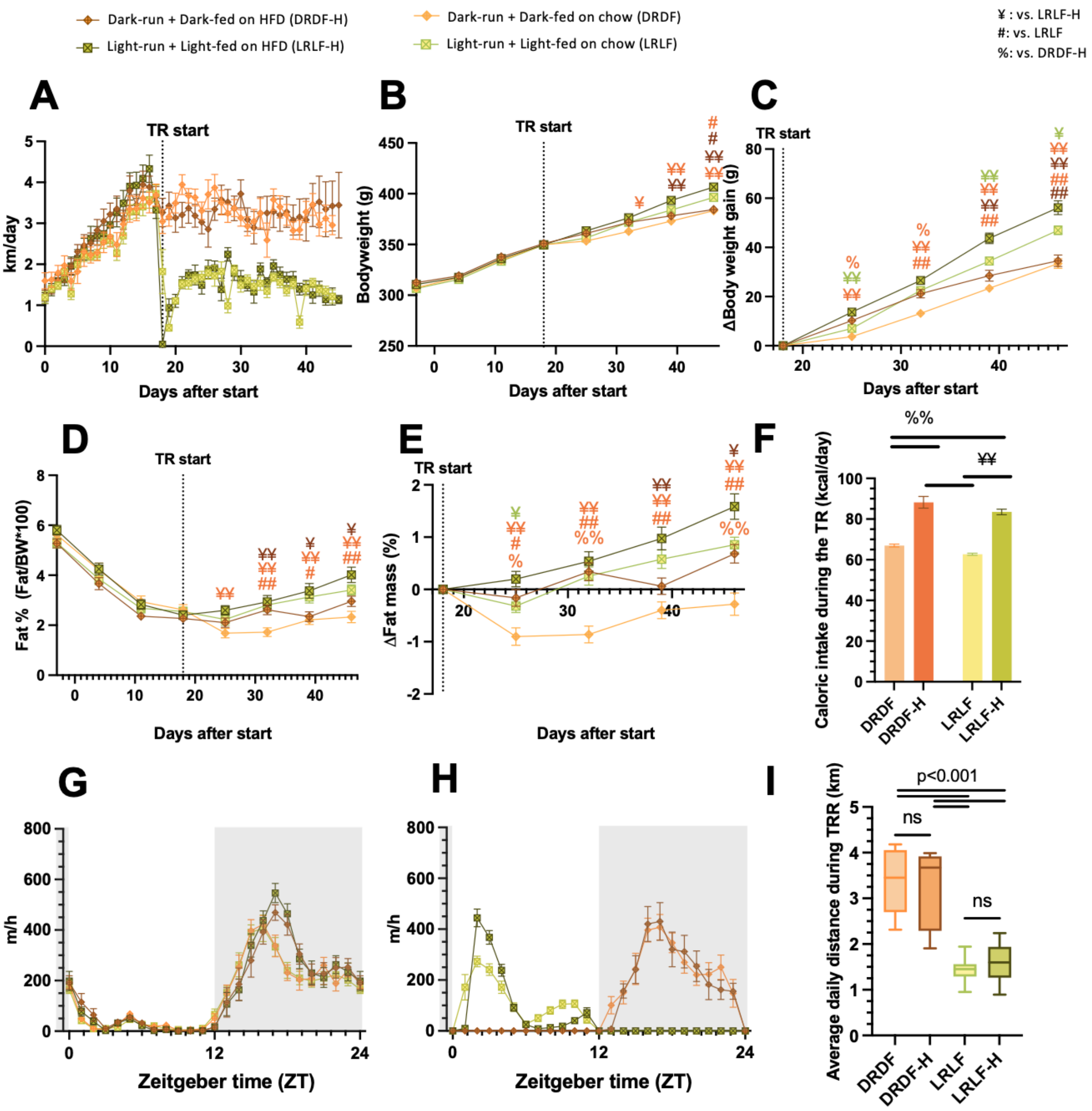

Supplemental Figure 1 (S1).

Comparison between time-restricted wheel running and time-restricted feeding on high-fat diet (HFD) (Fig.1) and chow (Shiba et al., 2024) . **A:** The average daily running distance of each group during the experiment. **B:** Body weight development. **C:** Gain of body weight from the start of the time restriction. **D:** Fat mass in percentage of body weight. **E:** Gain of body fat from the start of the time restriction. **F:** Caloric intake during the time restriction. **G-H:** Daily running pattern during the baseline **(G)** and time-restricted phase **(H)** per animal. **I:** Total running distance of one week (which week?) during time restriction of dark running dark fed chow (DRDF), dark running dark fed with high fat diet (DRDF-H), light running light fed chow (LRLF), and light running light fed with high fat diet (LRLF-H). Whiskers present maximum and minimum values. Dark running dark fed group on high fat diet (DRDF-H, in orange with brown frame): n= 24, Dark running dark fed group on chow diet (DRDF, in light orange with orange frame): n= 24, Light running light fed group on high fat diet (LRLF-H, in yellow green with olive frame): n= 24, Light running light fed group on chow diet (LRLF-H, in light yellow with light green frame): n= 24. Data are presented as the mean ± SEM. Significant difference from LRLF-H (¥), LRLF from (#), or from DRDF-H (%) compared to the groups of color code. ¥ or # or %: P < 0.05, ¥¥ or ## or %%: P < 0.01 by one-way ANOVA or mixed-effect analysis followed by Tukey HSD *post-hoc* test. TR: time restriction

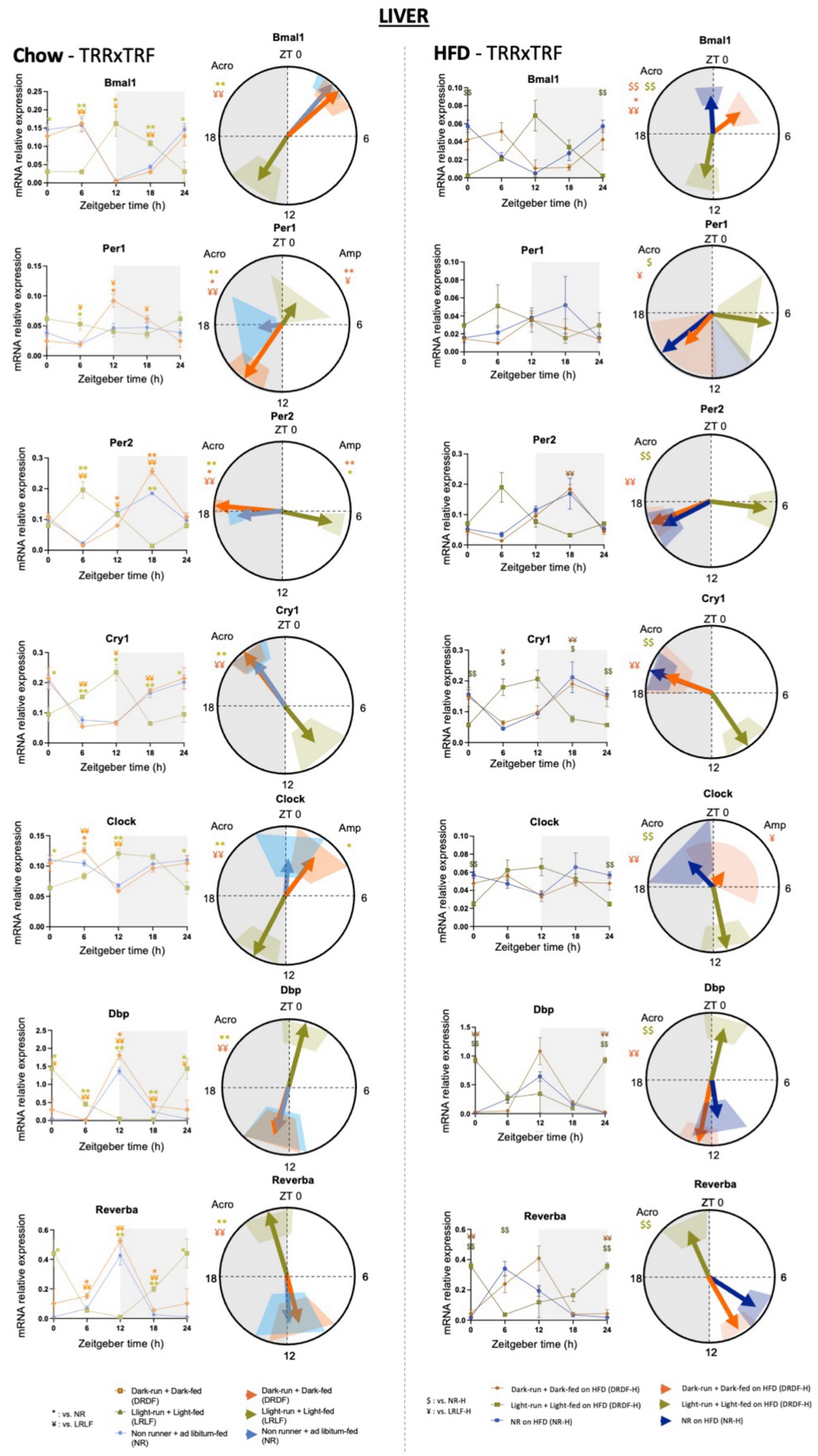

Supplemental Figure 2 (S2).

The comparison between liver clock gene expression patterns during combined time-restricted feeding and time-restricted running conditions when on a chow or high-fat diet.

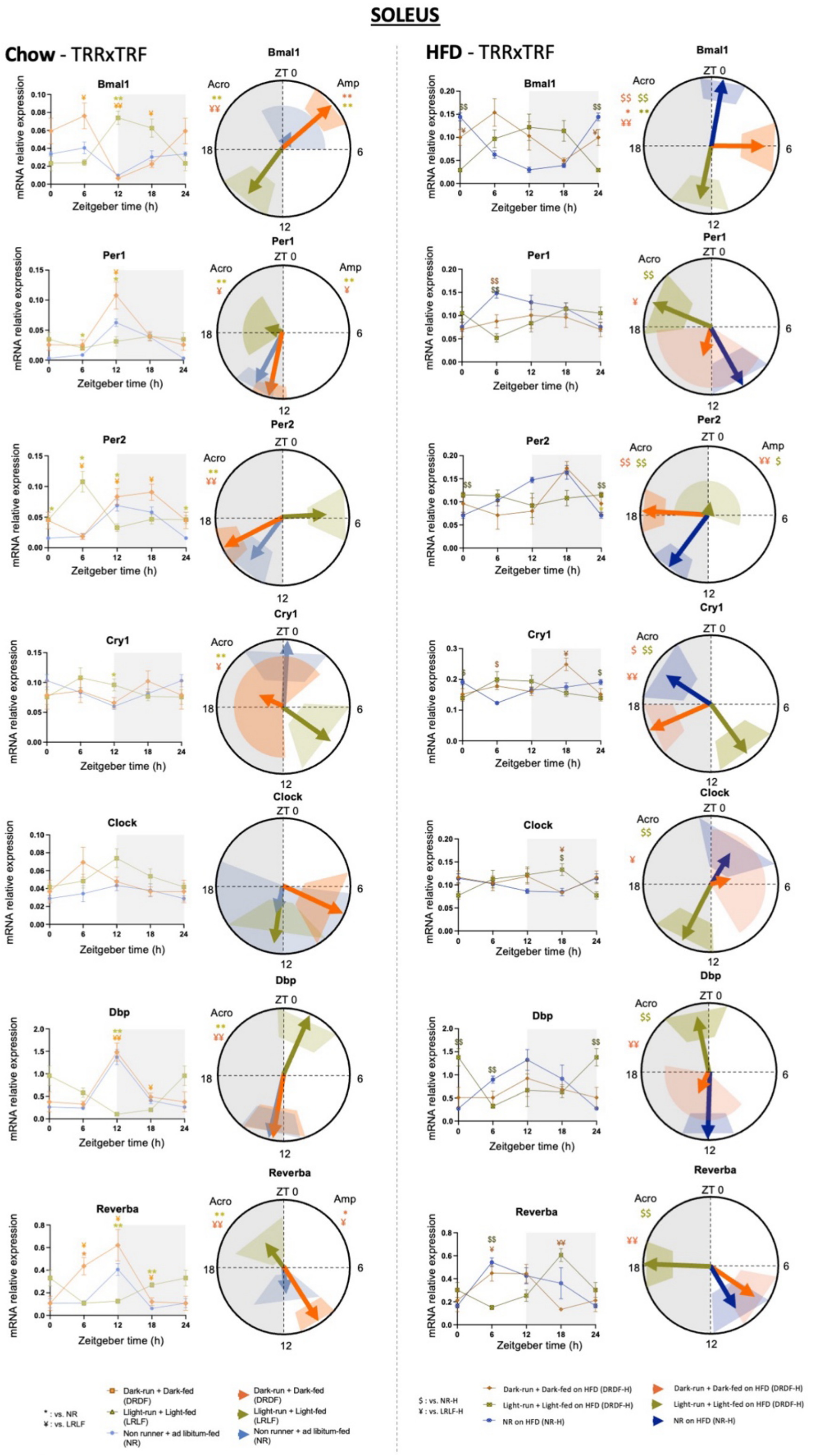

Supplemental Figure 3 (S3).

The comparison between soleus clock gene expression patterns during combined time-restricted feeding and time-restricted running conditions when on a chow or high-fat diet.

Supplemental Table S1
Summary of PCR primers

House-keeping gene	Symbol	Forward primer	Reverse primer	Used for
Beta2 macroglobulin	B2m	TGACCGTGATCTTTCTGGTGC	GCTTCCCATTCTCCGGTGG	liver
Beta-actin	Actb	ACAACCTTCTTGCAGCTCCTC	CTGACCCATACCCACCATCAC	muscle
Cyclophilin	Ppib	ATGTGGTCTTTGGGAAGGTG	GAAGGAATGGTTTGATGGGT	muscle
Glyceraldehyde-3-phosphate dehydrogenase	Gapdh	TGAACGGGAAGCTCACTGG	TCCACCACCCTGTTG CTGTA	liver
Hypoxanthine phosphoribosyltransferase 1	Hprt1	GCAGTACAGCCCCAAAATGG	AACAAAGTCTGGCCTGTATCCAA	muscle
TATA box-binding protein	Tbp	TTCGTGCCAGAAATGCTGAA	TGCACACCATTTTCCCAGAAC	liver
Sarcoplasmic/endoplasmic reticulum calcium ATPase 2	Serc2a	ATGGACGAGACGCTCAAGTT	GAAGCGGTTACTCCAGTATTGC	muscle
**Clock gene**	**Symbol**			
Brain and muscle arnt-like	Bmal1 (Arntl)	CCGATGACGAACTGAAACACCT	TGCAGTGTCCGAGGAAGATAGC	Both
circadian locomotor output cycles kaput	Clock	CGATCACAGCCCAACTCCTT	TTGCAGCTTGAGACATCGCT	Both
Cryptochrome Circadian Regulator 1	Cry1	AAGTCATCGTGCGCATTTCA	TCATCATGGTCGTCGGACAGA	Both
Period circadian regulator 1	Per1	CGCACTTCGGGAGCTCAAACTTC	GTCCATGGCACAGGGCTCACC	Both
Period circadian regulator 2	Per2	CACCCTGAAAAGAAAGTGCGA	CAACGCCAAGGAGCTCAAGT	Both
Rev-Erb alpha	Nr1d1	ACAGCTGACACCACCCAGATC	CATGGGCATAGGTGAAGATTTCT	Both
D site of the albumin promoter (albumin D-box) binding protein	Dbp	CCTTTGAACCTGATCCGGCT	TGCCTTCTTCATGATTGGCTG	Both
**Metabolic gene**	**Symbol**			
Perilipin-5	Plin5	GGCTACTTTGTGCGTCTGGGATC	CATCTCCTGGGTGCGGTGTTTG	muscle

Supplemental Table S2
Summary of mixed-effects analysis results of Fig. 1B-E, Supplemental Fig.1B-E. P-values followed by F-values in (brackets).

		Time	Group	Interaction
Fig. 1	B	**<0.0001** (2130)	**<0.0001** (54.37)	**<0.0001** (40)
	C	**<0.0001** (747.6)	**<0.0001** (19.42)	**<0.0001** (16.54)
	D	**<0.0001** (44.73)	**<0.0001** (75.21)	**<0.0001** (94.95)
	E	**<0.0001** (86.39)	**<0.0001** (17.49)	**<0.0001** (12.83)
Supplemental Fig. 1	B	**<0.0001** (1884)	0.0741 (2.388)	**<0.0001** (10.77)
	C	**<0.0001** (914.4)	**<0.0001** (26.59)	**<0.0001** (16.28)
	D	**<0.0001** (203.8)	**0.0389** (2.905)	**<0.0001** (5.818)
	E	**<0.0001** (38.03)	**<0.0001** (17.73)	**<0.0001** (7.087)

Significant values **in bold letters.**

Supplemental Table S3
Summary of mixed-effects analysis results of Fig. 2A, 3A and 4A. P-values followed by F-values in (brackets).

	Liver			Soleus		
**Genes**	**Time**	**Group**	**Interaction**	**Time**	**Group**	**Interaction**
Bmal1	0,4321 (0.8878)	0,3049 (1.2787)	**<0,0001** (12.81)	0,1339 (2.024)	0,0802 (2.614)	**<0,0001** (11.54)
Clock	0,0652 (2.679)	0,4461 (0.8522)	**0,0001** (5.141)	0,7558 (0.3549)	0,6709 (0.4100)	**<0,0001** (6.484)
Per1	0,4833 (0.7822)	0,3917 (0.9502)	0,4301 (1.019)	0,1837 (1.760)	**0,0443** (3.257)	**0,0042** (3.138)
Per2	**0,0029** (0.6685)	0,6830 (0.3912)	**<0,0001** (10.17)	**0,0038** (6.263)	0,7887 (0.2411)	**0,0014** (3.773)
Cry1	**0,0391** (3.253)	0,4170 (0.8853)	**<0,0001** (10.24)	**0,0425** (3.361)	0,4818 (0.7669)	**<0,0001** (7.945)
Reverb-α	**0,0054** (6.511)	**0,0070** (5.309)	**<0,0001** (16.21)	**0,0102** (4.518)	0,7302 (0.3211)	**<0,0001** (6.774)
DBP	**0,0009** (13.25)	**0,0035** (8.436)	**<0,0001** (14.11)	0,2632 (1.385)	0,1658 (1.843)	**<0.0001** (5.030)
Plin5 (chow)				0,0783 (2.516)	**0,0004** (13.61)	0,4172 (1.040)
Plin5 (HFD)				0,2756 (1.348)	**0,0003** (14.18)	**<0,0001** (9.576)

Significant values **in bold letters.**

Supplemental Table S4
Summary of raw PCR clock gene values outputted by CosinorPy analysis for Fig. 2B and 3B.

Liver					Soleus				
Parameter	Gene	NR-H	DRDF-H	LRLF-H	Parameter	Gene	NR-H	DRDF-H	LRLF-H
Amplitude	Bmal1	0,027	0,025	0,032	Amplitude	Bmal1	0,064	0,052	0,053
	Cry1	0,088	0,067	0,090		Cry1	0,031	0,038	0,036
	Clock	0,013	0,007	0,023		Clock	0,018	0,010	0,029
	Per1	0,020	0,013	0,018		Per1	0,035	0,017	0,033
	Per2	0,076	0,089	0,079		Per2	0,051	0,051	0,010
	Dbp	0,299	0,481	0,387		Dbp	0,540	0,205	0,456
	Reverba	0,182	0,195	0,159		Reverba	0,178	0,192	0,233
Peak (ZT)	Bmal1	23,736	3,556	12,781	Peak (ZT)	Bmal1	0,721	6,102	12,733
	Cry1	19,315	19,411	9,706		Cry1	20,228	16,432	9,610
	Clock	21,045	2,498	11,195		Clock	2,114	4,997	13,742
	Per1	15,423	14,655	6,631		Per1	10,138	12,973	19,508
	Per2	16,192	16,673	6,486		Per2	14,414	18,210	0,817
	Dbp	11,435	12,781	0,913		Dbp	12,060	13,742	23,255
	Reverba	8,216	10,042	22,438		Reverba	9,946	8,312	18,114

Supplemental Table S5
p-values corresponding to the t-test for cosinor analysis results of Fig. 2B and 3B.
(Acrophase results are in the lower left part of the rectangle and Amplitude results in the upper right part of the rectangle.)
Significant values **in bold** letters displayed as either **<0.05** or **<0.01** depending on their p-values.

Liver			NR-H	DRDF-H	LRLF-H
acrophase	Bmal1	NR-H		0,805029	0,436961
		DRDF-H	**<0.01**		0,436961
		LRLF-H	**<0.01**	**<0.01**	
	Cry1	NR-H		0,538251	0,933669
		DRDF-H	9,41E-01		0,538251
		LRLF-H	**<0.01**	**<0.01**	
	Clock	NR-H		0,501973	0,288962
		DRDF-H	1,07E-01		**<0.05**
		LRLF-H	**<0.01**	**<0.01**	
	Per1	NR-H		0,768164	0,913977
		DRDF-H	0,800949		0,768164
		LRLF-H	**<0.05**	**<0.05**	
	Per2	NR-H		0,749122	0,875364
		DRDF-H	5,17E-01		0,749122
		LRLF-H	**<0.01**	**<0.01**	
	Dbp	NR-H		0,24896	0,572687
		DRDF-H	3,35E-01		0,572687
		LRLF-H	**<0.01**	**<0.01**	
	Reverba	NR-H		0,81157	0,81157
		DRDF-H	**P<0.05**		0,81157
		LRLF-H	**<0.01**	**<0.01**	

Soleus			NR-H	DRDF-H	LRLF-H
acrophase	Bmal1	NR-H		0,975694	0,975694
		DRDF-H	**<0.01**		0,975694
		LRLF-H	**<0.01**	**<0.01**	
	Cry1	NR-H		0,836482	0,836482
		DRDF-H	**<0.05**		0,876973
		LRLF-H	**<0.01**	**<0.01**	
	Clock	NR-H		0,571382	0,313838
		DRDF-H	4,02E-01		0,313838
		LRLF-H	**<0.01**	**<0.05**	
	Per1	NR-H		0,827944	0,892044
		DRDF-H	4,44E-01		0,827944
		LRLF-H	**<0.01**	**<0.05**	
	Per2	NR-H		0,991132	**<0.01**
		DRDF-H	**<0.01**		**<0.05**
		LRLF-H	**<0.01**	0,236362	
	Dbp	NR-H		0,263371	0,744249
		DRDF-H	7,69E-01		0,587744
		LRLF-H	**<0.01**	**<0.01**	
	Reverba	NR-H		0,880052	0,880052
		DRDF-H	2,77E-01		0,880052
		LRLF-H	**<0.01**	**<0.01**	

Supplemental Table S6

The overview of cosinor results. **A:** Summary of raw Plin5 values outputted by CosinorPy analysis for Fig. 4A and B. **B:** p-values corresponding to the t-test for cosinor analysis results of Fig. 4A and B. (Acrophase results are in the lower left part of the rectangle and Amplitude results in the upper right part of the rectangle.)

Significant values **in bold** letters displayed as either **<0.05** or **<0.01** depending on their p-values..

A

**Chow**	NR	DRDF	LRLF
Amplitude	0,020	0,018	0,023
Peak(ZT)	6,102	6,775	1,538

**HFD**	NR-H	DRDF-H	LRLF-H
Amplitude	0,027	0,045	0,018
Peak(ZT)	2,883	9,946	17,393

B

chow	amplitude			
		NR	DRDF	LRLF
acrophase	NR		0.869426	0.869426
	DRDF	0.774985		0.869426
	LRLF	**<0.05**	**<0.05**	

HFD	amplitude			
		NR-H	DRDF-H	LRLF-H
acrophase	NR-H		0,496406	0,779129
	DRDF-H	**<0.01**		0,315314
	LRLF-H	**<0.01**	**<0.01**	
